# Electrophysiological Characterization of Sex-Dependent Hypnosis by an Endogenous Neuroactive Steroid Epipregnanolone

**DOI:** 10.3390/biom15071033

**Published:** 2025-07-17

**Authors:** Tamara Timic Stamenic, Ian Coulter, Douglas F. Covey, Slobodan M. Todorovic

**Affiliations:** 1 Department of Anesthesiology, University of Colorado, Anschutz Medical Campus, Aurora, CO 80045, USA; itcoulter@msn.com; 2 Department of Developmental Biology, Washington University School of Medicine, Saint Louis, MO 63101, USA; dcovey@wustl.edu; 3 Taylor Family Institute for Innovative Psychiatric Research, Washington University School of Medicine, Saint Louis, MO 63101, USA; 4 Neuroscience and Pharmacology Graduate Program, University of Colorado, Anschutz Medical Campus, Aurora, CO 80045, USA

**Keywords:** neuroactive steroids, hypnosis, electroencephalogram, sex differences, general anesthetics

## Abstract

Neuroactive steroids (NAS) have long been recognized for their hypnotic and anesthetic properties in both clinical and preclinical settings. While sex differences in NAS sensitivity are acknowledged, the underlying mechanisms remain poorly understood. Here, we examined sex-specific responses to an endogenous NAS epipregnanolone (EpiP) in wild-type mice using behavioral assessment of hypnosis (loss of righting reflex, LORR) and in vivo electrophysiological recordings. Specifically, local field potentials (LFPs) were recorded from the central medial thalamus (CMT) and electroencephalogram (EEG) signals were recorded from the barrel cortex. We found that EpiP-induced LORR exhibited clear sex differences, with females showing increased sensitivity. Spectral power analysis and thalamocortical (TC) and corticocortical (CC) phase synchronization further supported enhanced hypnotic susceptibility in female mice. Our findings reveal characteristic sex-dependent effects of EpiP on the synchronized electrical activity in both thalamus and cortex. These results support renewed exploration of endogenous NAS as clinically relevant anesthetic agents.

## 1. Introduction

Neuroactive steroids (NAS) are exogenous or endogenous steroids with direct effects on the central nervous system [1]. Epipregnanolone (EpiP, also known as 3β-hydroxy-5β-pregnan-20-one) is a naturally occurring NAS synthesized mostly in the periphery from progesterone [1,2]. It belongs to the family of pregnane steroids and is closely related chemically and functionally to other NASs, such as allopregnanolone [2,3]. The orientation of the hydroxyl group at the C3 position on the steroid backbone significantly influences the biological activity of NASs by altering their interactions with various ion channels and receptors [4,5]. EpiP, as a 3β-hydroxy NAS, primarily acts as an antagonist or negative modulator of the γ-aminobutyric acid type A (GABA_A_) receptor, in contrast to allopregnanolone, a 3α-hydroxy NAS, which is a potent positive modulator of this receptor [6,7]. Furthermore, it has been demonstrated that 3β-hydroxy NASs reduce the stimulating effect of 3α-hydroxy NASs on γ-aminobutyric acid (GABA) currents [4,8]. Interestingly, in rats, several 3β-hydroxysteroids, including EpiP, were found to weaken the enhancement of GABA currents by allopregnanolone [9]. However, when tested alone (without allopregnanolone), they acted as weak positive modulators of GABA currents [9]. Specifically, a recent study revealed that EpiP can act as a mild positive modulator with high affinity for GABA_A_ receptors alone, but with at least 4 times less potency than allopregnanolone [4]. This makes EpiP relatively unique, as it can potentially counteract or modulate the effects of other NASs. The modulatory effects of EpiP on GABA_A_ receptors influence neuronal excitability, contributing to its potential therapeutic roles in conditions like epilepsy, mood disorders, and substance use disorders, particularly in modulating the effects of other NASs [7].

In addition to the above studies, we recently used mouse genetics to show that EpiP may have significant hypnotic/sedative properties at least partly by inhibiting different isoforms of voltage-gated T-type calcium channels [3]. Additionally, we demonstrated that a synthetic EpiP analogue 3β-OH ((3β,5β,17β)-3-hydroxyandrostane-17-carbonitrile) displays strong sedative/hypnotic properties in rat pups, adult rats and mice [10,11,12,13]. We also recently reported sex-dependent hypnotic effects with both endogenous neurosteroid, allopregnanolone, and its synthetic analogue, alphaxalone, in mice, revealing that females are more sensitive using behavioral testing and in vivo electrophysiology [14]. Here, we set out to investigate possible sex differences in the EpiP-mediated hypnotic effect in mice using loss of righting reflex (LORR) and in vivo electrophysiology (local field potential (LFP) from intralaminar thalamus (central medial nucleus—CMT) and electroencephalogram (EEG) from barrel cortex).

## 2. Materials and Methods

All experimental procedures involving mice were approved by the Institutional Animal Care and Use Committee (IACUC; protocol number 0159) at the University of Colorado Anschutz Medical Campus (Aurora, CO, USA) and were carried out in accordance with the NIH Guide for the Care and Use of Laboratory Animals. Every effort was made to minimize animal discomfort and to use the fewest animals necessary to ensure statistically valid results. Adult male and female C57BL/6J wild-type mice (3–4 months old; Jackson Laboratory, Bar Harbor, ME, USA) were used for in vivo EEG recordings and behavioral experiments. Mice were housed under a 14:10 h light–dark cycle with free access to food and water. All experimental evaluations were conducted in a blinded fashion: although all animals received identical treatment, the investigators remained unaware of the animals’ sex during analysis.

NAS preparation

**Figure 1:**
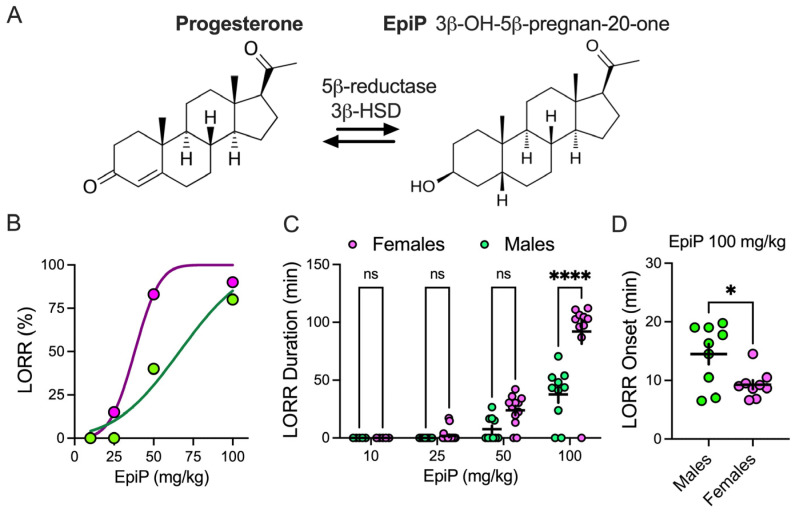
Neurosteroid synthesis and behavioral (LORR) assessments. (**A**) Schematic illustrating the synthesis of EpiP from progesterone via 5β-reductase and 3β-hydroxysteroid dehydrogenase (3β-HSD). (**B**) Dose–response curves showing the percentage of animals exhibiting loss of righting reflex (LORR) following EpiP administration. Note the marked rightward shift in the dose–response curve for the male EpiP group (green). (**C**) Duration of LORR at varying EpiP doses (11–20 mice per group); mixed-effect model (REML): sex F_(1,74)_ = 23.01, *p* < 0.001, dose F_(3,74)_ = 74.12, *p* < 0.001, interaction F_(3,74)_ = 12.52, *p* < 0.001; Sidak’s post hoc results indicated in the figure. (**D**) LORR onset following 100 mg/kg EpiP (9 mice per group); unpaired two-tailed t-test: t_(16)_ = 2.74, *p* = 0.003. Green = males, pink = females, * *p* < 0.05, **** *p* < 0.001.

EpiP (Figure 1A, Steraloids INC., Newport, RI, USA) was dissolved in stock solution of 15% cyclodextrin (Santa Cruz Biotechnology, Dallas, TX, USA) to yield the desired concentration for intraperitoneal (i.p.) injections. All doses of EpiP solution were prepared on the same day of the experiment. Mice were weighed and injected with the appropriate volume of EpiP to achieve the desired dose for behavioral experiments (ranging from 10 mg/kg to 100 mg/kg) and in vivo recordings (100 mg/kg). Because higher EpiP doses consistently induced LORR (Figure 1), we used heating pads and pulse oximetry in all experiments to assure normothermia and normal oxygenation of treated mice. If oxygen saturation dropped below the normal threshold (>90–95%) during an experiment, the recording from that animal would be excluded from the analysis. All animals maintained normal oxygenation in our experiments.

Loss of the righting reflex (LORR)

LORR is a behavioral indication of loss of consciousness, and it is a common technique used in preclinical research to assess the hypnotic effects of drugs or other interventions in rodents [15]. It involves placing an animal on its back and observing if it can flip over to regain a ventral (belly down) position [15]. After baseline habituation to the testing chamber (30 min), mice received i.p. injections of EpiP and were gently placed on their backs every 30 s [3,14]. A mouse is considered to have lost the righting reflex if it fails to right itself within 30 s when placed in the supine/ventral position. We noted the onset and duration of LORR in all tested mice.

Electrophysiological Recordings and Data Acquisition

Simultaneous, time-synchronized video and LFP recordings were acquired using a Pinnacle Technology system (Pinnacle Technology Inc., Lawrence, KS, USA). LFP signals were amplified 100-fold, digitized at a sampling rate of 2000 Hz, and bandpass-filtered between 0.5 and 500 Hz before being saved for offline analysis. Electrodes included a tungsten depth electrode targeting the central medial thalamic nucleus (CMT; coordinates: AP −1.35 mm, ML 0 mm, DV −3.6 mm) and two screw-type cortical electrodes (AP −1 mm, ML ±3 mm). Surgeries were conducted under 2.5 vol% isoflurane anesthesia. Postoperative analgesia with Banamine^®^ (flunixin, 2.5 mg/kg i.p.) was administered immediately after surgery and every 24 h for 48 h. Recordings were performed 7–10 days post-surgery in male and female wild-type (WT) mice, housed in custom recording chambers (40 cm × 40 cm × 42 cm). Baseline LFPs were recorded for at least 60 min before EpiP injection. For spectral analysis, 5 min epochs during baseline (wake episodes) and 25–30 min after EpiP injection were extracted and analyzed. Spectral analysis was performed using LabChart 8, Brainstorm and Origin 2018. Relative power (%) was calculated for standard frequency bands: delta (0.5–4 Hz), theta (4–8 Hz), alpha (8–13 Hz), beta (13–30 Hz), and low gamma (0–50 Hz). Power density (μV^2^/Hz) and full-range spectrograms (0.5–50 Hz) were also evaluated. Additionally, thalamocortical (TC) and corticocortical (CC) synchronization was analyzed, as well as phase–amplitude coupling (PAC; modulation index (MI)). Mice were anesthetized with ketamine (100 mg/kg i.p.) and isoflurane. Electrode placement was verified via electrolytic lesioning (5 μA for 1 s, repeated 5 times); then, the mice were perfused transcardially with ice-cold 0.1 M phosphate buffer containing 1% potassium ferrocyanide. Brains were fixed in 4% paraformaldehyde for 48 h, sectioned at 100 μm using a vibratome (Leica VT1200S, Leica Biosystems Nussloch GmbH, Nussloch, Germany), and imaged under a Zeiss stereoscope using Zen Blue 2.3 software. If deep electrode conformation showed poor deep electrode placement, the thalamic recording was excluded from analysis. Based on this criterion, two thalamic recordings, one female and one male, were excluded from analysis.

Data Analysis

Statistical analyses were conducted using two-way repeated measures (RM) ANOVA for in vivo datasets and mixed-effect models (REML) for behavioral data, along with unpaired two-tailed Student t-tests when appropriate. When a significant interaction between factors was detected in the two-way RM ANOVA, Sidak’s multiple-comparison test was applied. Statistical significance was defined as *p* < 0.05. All statistical and graphical analyses were carried out using GraphPad Prism version 8.00 (GraphPad Software, La Jolla, CA, USA) and Origin 2018 (OriginLab, Northampton, MA, USA). EEG/LFP signals were analyzed with LabChart 8 software (AD Instruments, Dunedin, New Zealand). The EEG frequency spectrum was segmented into delta (0.5–4 Hz), theta (4–8 Hz), alpha (8–13 Hz), beta (13–30 Hz), and low gamma (30–50 Hz) bands. Power density, total power, and relative power spectra were calculated using LabChart 8 (ADInstruments Inc., Colorado Springs, CO, USA). For additional EEG/LFP analysis we used the Brainstorm 2024 software package implemented in MATLAB 2024 [16]. We calculated MI for PAC and thalamocortical (TC) and corticocortical (CC) phase locking values (PLVs) for functional connectivity. The MI detected phase–amplitude coupling between two frequencies: the delta (phase modulating) and low gamma (amplitude-modulated) frequency bands. The PLV was used as a non-directed functional connectivity metric to capture interdependence between two signals: TC (CMT and cortex) and CC(right and left cortex) [17]. All data were presented as Mean ± Standard Error.

## 3. Results

### 3.1. Sex Differences in LORR

Figure 1A shows biosynthesis of the EpiP from progesterone; EpiP is derived from progesterone by reduction at the 5- and 3-positions of the steroid A-ring. The 5β-reductase and 3β-hydroxysteroid dehydrogenase (HSD) are responsible for EpiP synthesis [1,2]. Dose–response curves for the percent of animals with LORR showed a pronounced rightward shift in male mice injected with EpiP, indicating lower sensitivity in comparison to females (EpiP ED_50_: females 40.04 mg/kg; males 67.75 mg/kg; Figure 1B). We examined the effect of escalating EpiP doses on the duration of LORR in mice of both sexes (Figure 1C). We found that EpiP showed a sex-dependent effect with the highest dose (100 mg/kg) in LORR duration (Figure 1C). Specifically, female mice had about two-fold longer LORR duration (Figure 1C). Additionally, male mice injected with 100 mg/kg EpiP needed more time for LORR to occur in comparison to female animals (Figure 1D).

### 3.2. Sex Differences in Spectral Characteristics

**Figure 2:**
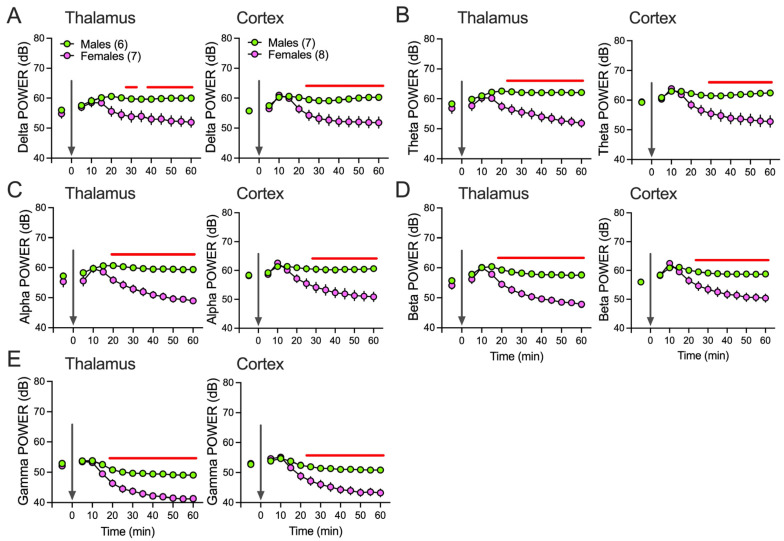
Power changes under EpiP over time. Total thalamic (**left**) and cortical (**right**) delta (**A**), theta (**B**), alpha (**C**), beta (**D**) and low gamma (**E**) power during wake state and after 100 mg/kg EpiP. Statistical analysis: two-way RM ANOVA for thalamic delta power: interaction F_(12,132)_ = 8.44, *p* < 0.001, time F_(12,132)_ = 5.63, *p* < 0.001, sex F_(1,11)_ = 7.71, *p* = 0.018; two-way RM ANOVA for cortical delta power: interaction F_(12,156)_ = 16.16, *p* < 0.001, time F_(12,156)_ = 16.00, *p* < 0.001, sex F_(1,13)_ = 9.4, *p* = 0.009; two-way RM ANOVA for thalamic theta power: interaction F_(12,132)_ = 10.92, *p* < 0.001, time F_(12,132)_ = 6.90, *p* < 0.001, sex F_(1,11)_ = 15.28, *p* = 0.002; two-way RM ANOVA for cortical theta power: interaction F_(12,156)_ = 16.38, *p* < 0.001, time F_(12,156)_ = 16.78, *p* < 0.001, sex F_(1,13)_ = 9.82, *p* = 0.008; two-way RM ANOVA for thalamic alpha power: interaction F_(12,132)_ = 10.74, *p* < 0.001, time F_(12,132)_ = 11.94, *p* < 0.001, sex F_(1,11)_ = 31.11, *p* < 0.001; two-way RM ANOVA for cortical alpha power: interaction F_(12,156)_ = 18.11, *p* < 0.001, time F_(12,156)_ = 19.33, *p* < 0.001, sex F_(1,13)_ = 10.74, *p* = 0.006; two-way RM ANOVA for thalamic beta power: interaction F_(12,132)_ = 10.84, *p* < 0.001, time F_(12,132)_ = 21.72, *p* < 0.001, sex F_(1,11)_ = 45.90, *p* < 0.001; two-way RM ANOVA for cortical beta power: interaction F_(12,156)_ = 16.70, *p* < 0.001, time F_(12,156)_ = 28.58, *p* < 0.001, sex F_(1,13)_ = 12.35, *p* = 0.004; two-way RM ANOVA for thalamic low gamma power: interaction F_(12,132)_ = 10.61, *p* < 0.001, time F_(12,132)_ = 53.46, *p* < 0.001, sex F_(1,11)_ = 35.41, *p* < 0.001; two-way RM ANOVA for cortical low gamma power: interaction F_(12,156)_ = 16.87, *p* < 0.001, time F_(12,156)_ = 53.85, *p* < 0.001, sex F_(1,13)_ = 15.14, *p* = 0.002. Green—males; pink—females; number of mice per group is presented in figure; Sidak’s post hoc presented in figure as a red line representing statistical significance, arrow—EpiP injection.

Power changes and sex differences after EpiP over time (60 min) are presented in Figure 2. There was a statistically significant increase in both thalamic and cortical power in male animals at all analyzed frequencies during a period of 30–60 min after EpiP injection in comparison to female mice: delta (Figure 2A), theta (Figure 2B), alpha (Figure 2C), beta (Figure 2D) and low gamma (Figure 2E).

**Figure 3:**
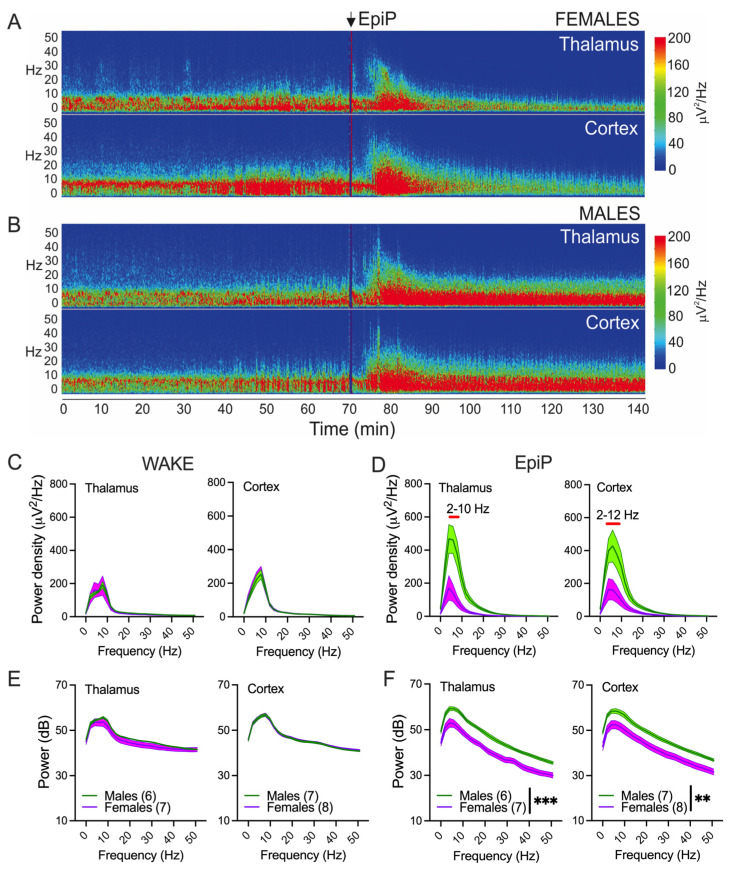
Spectral changes and sex differences under EpiP. (**A**) Representative thalamic (**upper panel**) and cortical (**lower panel**) power density heat maps in female animal showing decrease in power density after EpiP-induced LORR. (**B**) Representative thalamic (**upper panel**) and cortical (**lower panel**) power density heat maps in male animal showing increase in power density after EpiP-induced LORR. (**C**) Thalamic (**left panel**) and cortical (**right panel**) power densities during wake state. (**D**) Thalamic (**left panel**) and cortical (**right panel**) power densities 25–30 min after injections of EpiP. Female mice had reduction in 2–10 Hz thalamic and 2–12 Hz cortical power densities in comparison to male mice under EpiP; two-way RM ANOVA for thalamic power density: interaction F_(26,286)_ = 8.81, *p* < 0.001, frequency F_(26,286)_ = 32.0, *p* < 0.001, sex F_(1,11)_ = 14.9, *p* = 0.003, Sidak’s post hoc presented in figure, where red line represents statistical significance; two-way RM ANOVA for cortical power density: interaction F_(26,338)_ = 5.736, *p* < 0.001, frequency F_(26,338)_ = 27.29, *p* < 0.001, sex F_(1,13)_ = 6.99, *p* = 0.020, Sidak’s post hoc presented in figure, where red line represents statistical significance. (**E**) Thalamic (**left panel**) and cortical (**right panel**) powers (dB) during wake state. (**F**) Thalamic (**left panel**) and cortical (**right panel**) powers (dB) 25–30 min after injections of EpiP. Female mice had smaller values of thalamic powers in comparison to the male mice under EpiP; two-way RM ANOVA for thalamic power: interaction F_(26,286)_ = 0.56, *p* = 0.961, frequency F_(26,266)_ = 384.2, *p* < 0.001, sex F_(1,11)_ = 22.49, *p* < 0.001; two-way RM ANOVA for cortical power: interaction F_(26,338)_ = 1.14, *p* = 0.288, frequency F_(26,338)_ = 1692, *p* < 0.001, sex F_(1,13)_ = 9.98, *p* = 0.007. Green—males; pink—females; number of mice per group is presented in figure, ** *p* < 0.01, *** *p* < 0.001.

The representative power density heat maps before and after EpiP (100 mg/kg) injection from the thalamus (up) and cortex (down) are presented in Figure 3A,B (female in Figure 3A and male in Figure 3B). No sex differences in thalamic or cortical power density were detected during wake periods before EpiP injection, either in the thalamus (Figure 3C,E left, respectively) or in the cortex (Figure 3C,E, right, respectively). However, 25–30 min after EpiP, male mice showed higher power densities in comparison to females between 2 and 10 Hz in the thalamus (Figure 3D left) and between 2 and 12 Hz in the cortex (Figure 3D right). Similarly, 25–30 min after EpiP, male animals had higher powers in all analyzed frequencies in the thalamus and the cortex in comparison to female mice (Figure 3F left and right, respectively). Furthermore, we observed the shift in peak power density towards slower oscillations after EpiP injection in both female and male mice in comparison to wake periods (from 8 Hz during wake to 4–6 Hz 25–30 min after EpiP injection).

### 3.3. Sex Differences in Brain Synchronization and Phase–Amplitude Coupling

**Figure 4:**
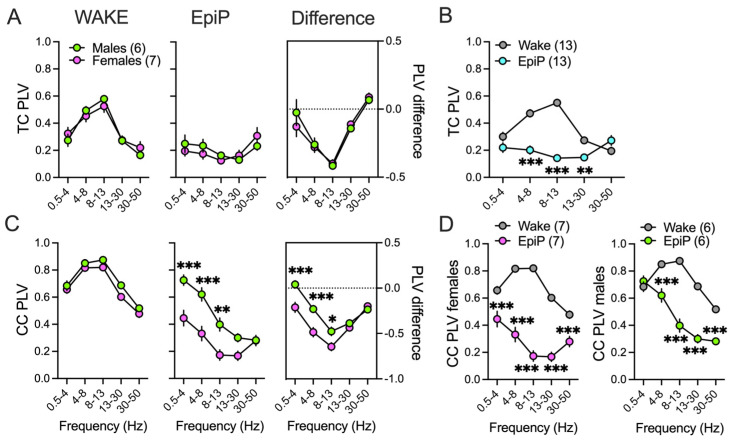
Sex-dependent differences in the cortical brain synchronization under EpiP. (**A**) Thalamocortical (TC) phase locking values (PLVs) during wake stateg (**left**), after EpiP (**middle**) and difference between EpiP and wake-state PLVs (**right**): sex differences were not observed. (**B**) Because there was no statistical significance between sexes during wake periods and after EpiP injection, both female and male data were combined for the statistical analysis: two-way RM ANOVA for EpiP: interaction F_(4,48)_ = 34.74, *p* < 0.001, frequency F_(4,48)_ = 12.58, *p* < 0.001, EpiP F_(1,12)_ = 98.25, *p* < 0.001. (**C**) Corticocortical (CC) PLVs during wake state (**left panel**), after EpiP (**middle panel**) and difference between EpiP and wake-state PLVs (**right panel**). Female animals had lower delta/theta/alpha CC PLVs in comparison to the male EpiP animals (**C middle panel**), two-way RM ANOVA: interaction F_(4,44)_ = 7.59, *p* < 0.001, frequency F_(4,44)_ = 48.21, *p* < 0.001, sex F_(1,11)_ = 15.23, *p* = 0.002, Sidak’s post hoc presented in figure. (**C right panel**) CC PLV difference showed lower values in female animals in delta/theta/alpha range; two-way RM ANOVA: interaction F_(4,44)_ = 9.4, *p* < 0.001, frequency F_(4,44)_ = 75.14, *p* < 0.001, sex F_(1,11)_ = 9.06, *p* = 0.012, Sidak’s post hoc presented in figure. (**D**) Because there was a sex-dependent effect on CC PLVs, data from female and male mice are analyzed separately: two-way RM ANOVA for EpiP females: interaction F_(4,24)_ = 28.63, *p* < 0.001, frequency F_(4,24)_ = 15.34, *p* < 0.001, EpiP F_(1,6)_ = 182.2, *p* < 0.001, Sidak’s post hoc presented in figure; two-way RM ANOVA for EpiP males: interaction F_(4,20)_ = 117.1, *p* < 0.001, frequency F_(4,20)_ = 198.6, *p* < 0.001, EpiP F_(1,5)_ = 52.11, *p* < 0.001, Sidak’s post hoc presented in figure. Green—males; pink—females; gray—wake; blue—EpiP; number of animals per group is presented in figure, * *p* < 0.05, ** *p* < 0.01, *** *p* < 0.001.

Consistent with the above findings, we did not observe sex differences during wake PLVs and in TC PLVs under EpiP (Figure 4A left, middle, respectively), as well as in analysis of difference between EpiP-induced and wake state PLVs (Figure 4A right), so we analyzed male and female data together (Figure 4B). Under EpiP we found a decrease in theta/alpha/beta TC PLVs in all animals (Figure 4B). Because female animals had lower delta, theta and alpha CC PLVs after EpiP in comparison to male mice (Figure 4C middle, right), we performed separate statistical analysis for female and male mice for CC PLVs (Figure 4D left, right, respectively). The female EpiP animals had a reduction in CC synchronization in all analyzed frequencies (Figure 4D left). By contrast, in male animals EpiP reduced the CC synchronization in all other frequencies but spared the delta range (Figure 4D right).

**Figure 5:**
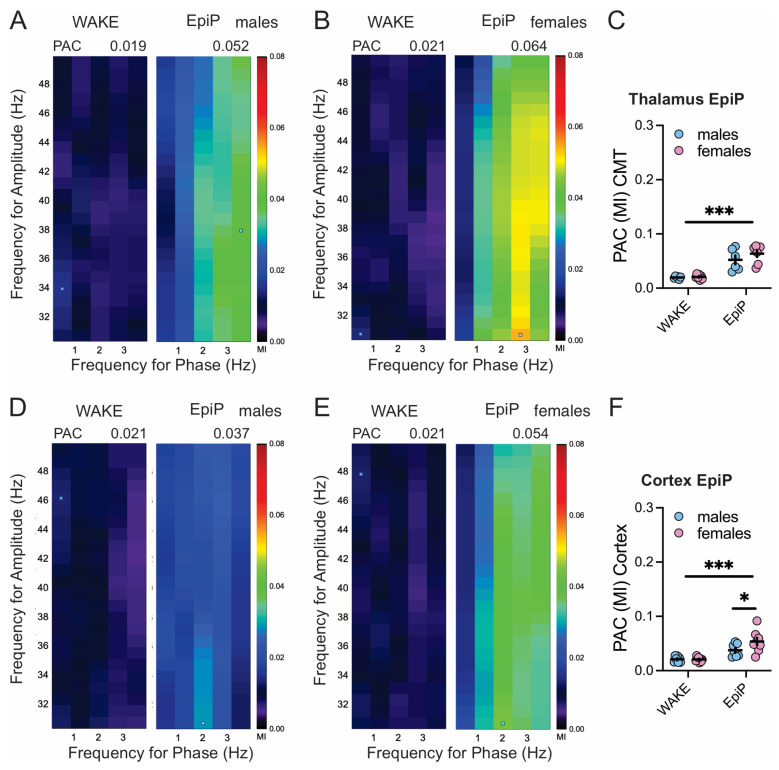
PAC under EpiP. Heat maps showing change in thalamic MI after EpiP in male (**A left -wake; A right-EpiP**) and female (**B left-wake; B right-EpiP**) mice across different phase modulating (0.5–4 Hz) and amplitude modulating (30–50 Hz) frequencies. (**C**) Averaged thalamic max PAC MI value during wake state and after injections of EpiP; two-way RM ANOVA: interaction F_(1,11)_ = 1.02, *p* = 0.334, EpiP F_(1,11)_ = 52.9, *p* < 0.001, sex F_(1,11)_ = 1.58, *p* = 0.23. Heat maps showing change in cortical MI after injections of EpiP in male (**D left-wake; D right-EpiP**) and female (**E left-wake; E right-EpiP**) mice across different phase modulating (0.5–4 Hz) and amplitude modulating (30–50 Hz) frequencies. (**F**) Averaged cortical max PAC MI value during wake state and after EpiP; two-way RM ANOVA: interaction F_(1,13)_ = 4.26, *p* = 0.05, EpiP F_(1,13)_ = 38.28, *p* < 0.001, sex F_(1,13)_ = 2.44, *p* = 0.142, Sidak’s post hoc presented in figure. Blue—males; pink—females; dot on heat maps is the max PAC MI; number of mice per group: thalamic: 6 males and 7 females; cortical: 7 males and 8 females; * *p* < 0.05, *** *p* < 0.001.

Figure 5 presents heat maps showing higher values of MI across phase modulating (0.5–4 Hz) and amplitude modulating (30–50 Hz) frequencies after injections of EpiP in male and female mice in the thalamus (Figure 5A,B) and the cortex (Figure 5D,E). We observed an increase in thalamic (Figure 5C) and cortical (Figure 5F) PAC after injections of EpiP in both females and males. Additionally, cortical PAC revealed lower delta/low gamma coupling in male mice (Figure 5F).

## 4. Discussion

Since the introduction of alphaxalone and related 3α-hydroxylated NASs in the 1970s, there has been growing interest in developing NAS-based anesthetics [7]. In this study, we demonstrate that an endogenous NAS EpiP produces a hypnotic/sedative effect, as measured by LORR, in both male and female WT mice. Notably, we identified significant sex differences in EpiP sensitivity: female mice exhibited greater susceptibility to EpiP-induced hypnosis. These findings align with our prior report showing similar sex differences in response to the synthetic EpiP analogue 3β-hydroxy (3β-OH), which we attributed to sex-specific peripheral metabolism into the more potent 3α-hydroxy GABA_A_ receptor-positive allosteric modulator [11,18]. A comparable metabolic pathway may underlie the heightened hypnotic sensitivity to EpiP observed in females, given its potential bioconversion to pregnanolone—a potent 3α-hydroxy GABA_A_-positive allosteric modulator [1,2]. This finding underscores the potential role of EpiP as a pro-drug that may be more effective in females than in males, likely due to sex-specific differences in its metabolism. Although this sex-dependent effect could have important implications for designing and interpreting clinical studies, further preclinical studies are needed to investigate this mechanism in detail.

In support of the above behavioral findings, our EEG recordings revealed additional sex-dependent effects of EpiP on brain oscillations. Following EpiP injection (100 mg/kg), female mice exhibited reductions in total cortical and thalamic power post-LORR, whereas male mice showed a progressive increase in the spectral power over time. In both the thalamus and cortex, spectral density shifted from theta frequencies (associated with wakefulness) toward delta activity, consistent with sedative/hypnotic states, which we also observed with allopregnanolone and alphaxalone [14]. Additionally, females displayed a pronounced reduction in overall power and power density, while males exhibited an increase, further supporting a sex-dependent divergence in response. Similarly to EpiP-treated females, we recently showed an overall power decrease after allopregnanolone in both male and female animals [14].

To examine large-scale network coordination, we analyzed TC and CC phase synchronization [19]. We found that EpiP treatment led to broad reductions in CC phase synchronization across all frequencies in female mice. Similarly, in males, administration of EpiP reduced synchronization at all frequencies except within the delta band, potentially explaining their partial resistance to LORR. The reduction in CC delta synchronization echoes prior studies with a common anesthetic agent such as propofol demonstrating reduced slow-wave coherence during anesthetic induction [20,21]. However, unlike propofol, which increases cortical alpha synchrony [22], we found that EpiP induced a widespread reduction in alpha-band synchronization in both TC and CC circuits. Also, in our experiments males displayed higher CC PLVs in delta, theta, and alpha bands compared to females—likely contributing to the observed sex differences in depth and duration of hypnosis.

We also analyzed PAC using the MI, focusing on delta phase–low gamma amplitude interactions. Previous studies have linked PAC patterns to cognitive function and anesthesia depth [23,24]. Consistent with prior work on propofol and sevoflurane [25,26,27], we found increased delta–low gamma PAC in both cortical and thalamic regions following EpiP administration. Notably, PAC was significantly higher in female mice, further indicating sex-dependent network-level differences during NAS-induced hypnosis.

These findings align with the well-established observation that, under basal conditions, circulating progesterone levels—the precursors for EpiP and allopregnanolone—are generally higher in females than in males [28,29,30,31]. Prior studies have further shown a positive correlation between progesterone and allopregnanolone concentrations in both plasma and brain tissue in rats [28,32]. Interestingly, in human studies, intravenous administration of allopregnanolone produced greater sedation in women than in men, despite lower serum levels, suggesting higher receptor sensitivity in women or possible differences in brain concentrations [33]. This sex-dependent response indicates that women’s GABA_A_ receptors may mediate sedation more effectively [33]. Beyond sedation, the effects of allopregnanolone on mood, anxiety, addiction, seizure activity, and neuroprotection similarly show sex-specific patterns, further supporting greater female sensitivity [34]. Sex differences in GABA receptor subunit expression have also been documented, with males exhibiting more low-affinity GABA binding sites but fewer high-affinity sites in various cortical regions compared to females [35]. Additionally, basal mRNA expression of key enzymes involved in the synthesis and metabolism of NASs is higher in females than in males in the cerebellum but not in the cortex, pointing to region-specific regulation [36,37]. Since many NASs enhance both phasic and tonic inhibitory synaptic transmission, these sex-specific differences in steroidogenesis and GABA receptor composition may help explain the differential vulnerability to certain brain disorders and partially account for our results [1]. Our findings with EpiP reveal a similar sex-dependent effect to that observed with allopregnanolone. Future research should further explore how these sex-dependent pathways interact with other modulators of GABAergic transmission and examine whether targeting NAS synthesis or specific receptor or ion channel subtypes could inform the development of sex-specific therapeutic strategies.

It is known that estrous cycle fluctuations cause dynamic changes in endogenous neuroactive steroid levels (e.g., progesterone → allopregnanolone), impacting GABAergic tone, anxiety, seizure thresholds [38]. Additionally, expression of δ-subunit-containing GABA_A_ receptors varies across the estrous cycle, influencing tonic inhibition and sensitivity to NASs [38]. Unfortunately, we did not specifically track estrous cycle in our experiments, and this can be one of the limitations of our study. Further, for our electrophysiological studies we chose a dose of 100 mg/kg which caused LORR in most of the male and female mice (Figure 1B). However, due to technical difficulties of performing both experiments at the same time, we did not assess LORR during in vivo EEG experiments and hence did not exclude animals based on behavioral criteria. Future experiments will need to address both limitations.

## 5. Conclusions

To our knowledge this is the very first comprehensive evaluation of electrophysiological signature of the thalamic and cortical activity during EpiP-indued sedation/hypnosis. Our findings demonstrate that EpiP induces robust hypnotic and EEG changes with clear sex-specific differences in behavioral sensitivity, brain oscillatory dynamics, and interregional synchronization showing females to be more sensitive to EpiP-mediated changes. While EpiP shares certain spectral features with classic GABA_A_-based anesthetics and 3α-hydroxy neuroactive steroids—particularly delta power enhancement—it also exhibits distinct sex-specific neurophysiological signatures. These unique properties may reflect differences in affinity for GABA_A_ receptors but also an effect on low-voltage-gated T-type calcium channels. Given the growing interest in NASs as anesthetics with favorable safety profiles and potentially fewer cognitive side effects, EpiP represents, together with alphaxalone and allopregnanolone, a promising candidate for further translational research. Our data support renewed investigation of EpiP and related NASs as viable alternatives to the traditional general anesthetics, with consideration for sex-specific efficacy and metabolism.

## Data Availability

Data will be made available from the corresponding author on request.

## Abbreviations

The following abbreviations are used in this manuscript:

EpiP	Epipregnanolone
LORR	Loss of righting reflex
EEG	Electroencephalogram
LFP	Local field potential
CMT	Central medial nucleus of thalamus
GABA_A_	γ-aminobutyric acid type A receptor
GABA	γ-aminobutyric acid type
MI	Modulation index
PAC	Phase–amplitude coupling
TC	Thalamocortical
CC	Corticocortical
PLV	Phase locking value
3β-HSD	3β-hydroxysteroid dehydrogenase
RM	Repeated measure
NAS	Neuroactive steroid

## References

[1] Reddy D.S. (2010). Neurosteroids: Endogenous Role in the Human Brian and Therapeutic Potentials. Prog. Brain Res..

[2] Penning T.M., Covey D.F. (2024). 5β-Dihydrosteroids: Formation and Properties. Int. J. Mol. Sci..

[3] Coulter I., Timic Stamenic T., Eggan P., Fine B.R., Corrigan T., Covey D.F., Yang L., Pan J.Q., Todorovic S.M. (2021). Different Roles of T-Type Calcium Channel Isoforms in Hypnosis Induced by an Endogenous Neurosteroid Epipregnanolone. Neuropharmacology.

[4] Bukanova J., Solntseva E., Kondratenko R., Kudova E. (2021). Epipregnanolone as a Positive Modulator of GABAA Receptor in Rat Cerebellar and Hippocampus Neurons. Biomolecules.

[5] Miller P.S., Scott S., Masiulis S., De Colibus L., Pardon E., Steyaert J., Aricescu A.R. (2017). Structural Basis for GABAA Receptor Potentiation by Neurosteroids. Nat. Struct. Mol. Biol..

[6] Lambert J.J., Belelli D., Peden D.R., Vardy A.W., Peters J.A. (2003). Neurosteroid Modulation of GABA A Receptors. Prog. Neurobiol..

[7] Maguire J.L., Mennerick S. (2024). Neurosteroids: Mechanistic Considerations and Clinical Prospects. Neuropsychopharmacology.

[8] Wang M., He Y., Eisenman L.N., Fields C., Zeng C.-M., Mathews J., Benz A., Fu T., Zorumski E., Steinbach J.H. (2002). 3β-Hydroxypregnane Steroids Are Pregnenolone Sulfate-like GABAA Receptor Antagonists. J. Neurosci..

[9] Strömberg J., Haage D., Taube M., Bäckström T., Lundgren P. (2006). Neurosteroid Modulation of Allopregnanolone and GABA Effect on the GABA-A Receptor. Neuroscience.

[10] Atluri N., Joksimovic S.M., Oklopcic A., Milanovic D., Klawitter J., Eggan P., Krishnan K., Covey D.F., Todorovic S.M., Jevtovic-Todorovic V. (2018). A Neurosteroid Analogue with T-Type Calcium Channel Blocking Properties Is an Effective Hypnotic, but Is Not Harmful to Neonatal Rat Brain. Br. J. Anaesth..

[11] Joksimovic S.M., Sampath D., Krishnan K., Covey D.F., Jevtovic-Todorovic V., Raol Y.H., Todorovic S.M. (2021). Differential Effects of the Novel Neurosteroid Hypnotic (3β,5β,17β)-3-Hydroxyandrostane-17-Carbonitrile on Electroencephalogram Activity in Male and Female Rats. Br. J. Anaesth..

[12] Timic Stamenic T., Manzella F.M., Maksimovic S., Krishnan K., Covey D.F., Jevtovic-Todorovic V., Todorovic S.M. (2022). Further Evidence That Inhibition of Neuronal Voltage-Gated Calcium Channels Contributes to the Hypnotic Effect of Neurosteroid Analogue, 3β-OH. Front. Pharmacol..

[13] Timic Stamenic T., Feseha S., Manzella F.M., Wallace D., Wilkey D., Corrigan T., Fiedler H., Doerr P., Krishnan K., Raol Y.H. (2021). The T-Type Calcium Channel Isoform Cav3.1 Is a Target for the Hypnotic Effect of the Anaesthetic Neurosteroid (3β,5β,17β)-3-Hydroxyandrostane-17-Carbonitrile. Br. J. Anaesth..

[14] Martin A., Coulter I., Cox R., Covey D.F., Todorovic S.M., Timic Stamenic T. (2025). Comparative Electrophysiological Study of Neuroactive Steroid-Induced Hypnosis in Mice: Sex and Drug-Specific Differences. Exp. Biol. Med..

[15] McCarren H.S., Moore J.T., Kelz M.B. (2013). Assessing Changes in Volatile General Anesthetic Sensitivity of Mice after Local or Systemic Pharmacological Intervention. J. Vis. Exp..

[16] Tadel F., Bock E., Niso G., Mosher J.C., Cousineau M., Pantazis D., Leahy R.M., Baillet S. (2019). MEG/EEG Group Analysis with Brainstorm. Front. Neurosci..

[17] Bastos A.M., Schoffelen J.M. (2016). A Tutorial Review of Functional Connectivity Analysis Methods and Their Interpretational Pitfalls. Front. Syst. Neurosci..

[18] Manzella F.M., Cabrera O.H., Wilkey D., Fine-Raquet B., Klawitter J., Krishnan K., Covey D.F., Jevtovic-Todorovic V., Todorovic S.M. (2023). Sex-Specific Hypnotic Effects of the Neuroactive Steroid (3β,5β,17β)-3-Hydroxyandrostane-17-Carbonitrile Are Mediated by Peripheral Metabolism into an Active Hypnotic Steroid. Br. J. Anaesth..

[19] Liang Z., Ren Y., Yan J., Li D., Voss L.J., Sleigh J.W., Li X. (2016). A Comparison of Different Synchronization Measures in Electroencephalogram during Propofol Anesthesia. J. Clin. Monit. Comput..

[20] Koskinen M., Seppänen T., Tuukkanen J., Yli-Hankala A., Jäntti V. (2001). Propofol Anesthesia Induces Phase Synchronization Changes in EEG. Clin. Neurophysiol..

[21] Wang K., Steyn-Ross M.L., Steyn-Ross D.A., Wilson M.T., Sleigh J.W. (2014). EEG Slow-Wave Coherence Changes in Propofol-Induced General Anesthesia: Experiment and Theory. Front. Syst. Neurosci..

[22] Nicolaou N., Georgiou J. (2014). Spatial Analytic Phase Difference of EEG Activity during Anesthetic-Induced Unconsciousness. Clin. Neurophysiol..

[23] Tsai F.F., Fan S.Z., Cheng H.L., Yeh J.R. (2019). Multi-Timescale Phase-Amplitude Couplings in Transitions of Anesthetic-Induced Unconsciousness. Sci. Rep..

[24] Pal D., Silverstein B.H., Sharba L., Li D., Hambrecht-Wiedbusch V.S., Hudetz A.G., Mashour G.A. (2017). Propofol, Sevoflurane, and Ketamine Induce a Reversible Increase in Delta-Gamma and Theta-Gamma Phase-Amplitude Coupling in Frontal Cortex of Rat. Front. Syst. Neurosci..

[25] Huang Y., Wu D., Bahuri N.F.A., Wang S., Hyam J.A., Yarrow S., FitzGerald J.J., Aziz T.Z., Green A.L. (2018). Spectral and Phase-Amplitude Coupling Signatures in Human Deep Brain Oscillations during Propofol-Induced Anaesthesia. Br. J. Anaesth..

[26] Dong K., Zhang D., Wei Q., Wang G., Huang F., Chen X., Muhammad K.G., Sun Y., Liu J. (2022). Intrinsic Phase–Amplitude Coupling on Multiple Spatial Scales during the Loss and Recovery of Consciousness. Comput. Biol. Med..

[27] Zakaria L., Desowska A., Berde C.B., Cornelissen L. (2023). Electroencephalographic Delta and Alpha Oscillations Reveal Phase-Amplitude Coupling in Paediatric Patients Undergoing Sevoflurane-Based General Anaesthesia. Br. J. Anaesth..

[28] Sze Y., Gill A.C., Brunton P.J. (2018). Sex-Dependent Changes in Neuroactive Steroid Concentrations in the Rat Brain Following Acute Swim Stress. J. Neuroendocrinol..

[29] Caruso D., D’Intino G., Giatti S., Maschi O., Pesaresi M., Calabrese D., Garcia-Segura L.M., Calza L., Melcangi R.C. (2010). Sex-Dimorphic Changes in Neuroactive Steroid Levels after Chronic Experimental Autoimmune Encephalomyelitis. J. Neurochem..

[30] Caruso D., Pesaresi M., Maschi O., Giatti S., Garcia-Segura L.M., Melcangi R.C. (2010). Effect of Short-and Long-Term Gonadectomy on Neuroactive Steroid Levels in the Central and Peripheral Nervous System of Male and Female Rats. J. Neuroendocrinol..

[31] Pisu M.G., Concas L., Siddi C., Serra M., Porcu P. (2022). The Allopregnanolone Response to Acute Stress in Females: Preclinical and Clinical Studies. Biomolecules.

[32] Vallée M., Rivera J.D., Koob G.F., Purdy R.H., Fitzgerald R.L. (2000). Quantification of Neurosteroids in Rat Plasma and Brain Following Swim Stress and Allopregnanolone Administration Using Negative Chemical Ionization Gas Chromatography/Mass Spectrometry. Anal. Biochem..

[33] van Broekhoven F., Bäckström T., van Luijtelaar G., Buitelaar J.K., Smits P., Verkes R.J. (2007). Effects of Allopregnanolone on Sedation in Men, and in Women on Oral Contraceptives. Psychoneuroendocrinology.

[34] Diviccaro S., Cioffi L., Falvo E., Giatti S., Melcangi R.C. (2022). Allopregnanolone: An Overview on Its Synthesis and Effects. J. Neuroendocrinol..

[35] Skilbeck K.J., Hinton T., Johnston G.A.R. (2008). Sex-Differences and Stress: Effects on Regional High and Low Affinity [3H]GABA Binding. Neurochem. Int..

[36] Giatti S., Diviccaro S., Garcia-Segura L.M., Melcangi R.C. (2019). Sex Differences in the Brain Expression of Steroidogenic Molecules under Basal Conditions and after Gonadectomy. J. Neuroendocrinol..

[37] Giatti S., Garcia-Segura L.M., Barreto G.E., Melcangi R.C. (2019). Neuroactive Steroids, Neurosteroidogenesis and Sex. Prog. Neurobiol..

[38] Samba Reddy D. (2017). Sex Differences in the Anticonvulsant Activity of Neurosteroids. J. Neurosci. Res..

